# From Raw to Cooked: Proximate Composition, Fatty Acids and Fat-Soluble Vitamins in Bluefish (*Pomatomus saltatrix*) from the Black Sea

**DOI:** 10.3390/foods15010055

**Published:** 2025-12-24

**Authors:** Veselina Panayotova, Katya Peycheva, Tatyana Hristova, Diana A. Dobreva, Tonika Stoycheva, Rositsa Stancheva, Stanislava Georgieva, Evgeni Andreev, Silviya Nikolova, Rouzha Pancheva, Albena Merdzhanova

**Affiliations:** 1 Department of Chemistry, Faculty of Pharmacy, Medical University of Varna, 9002 Varna, Bulgariaalbenamerdjanova@mu-varna.bg (A.M.); 2 Department of Information Technology, Faculty of Engineering, Nikola Vaptsarov Naval Academy, 9002 Varna, Bulgaria; 3 Department of Social Medicine and Healthcare Organization, Faculty of Public Health, Medical University of Varna, 9002 Varna, Bulgaria; 4 Department of Hygiene and Epidemiology, Faculty of Public Health, Medical University of Varna, 9002 Varna, Bulgaria

**Keywords:** bluefish, traditional cooking, nutritive value, fatty acids, vitamins, health-benefits

## Abstract

Bluefish (*Pomatomus saltatrix*) is an important Black Sea species; however, quantitative data on how traditional household cooking affects its nutritional composition remain limited. This study assessed the effects of grilling, pan-frying, and smoking on the proximate composition, fatty acid profile, fat-soluble vitamins, antioxidant pigments, and cholesterol content of bluefish. Cooking led to moisture reductions of 7–18%, accompanied by increased total lipid content (26–80%). Crude protein content decreased in grilled and smoked fish and increased in pan-fried samples. Pan-frying resulted in the largest reduction in long-chain n-3 PUFA, with reductions of approximately 25.2% for EPA and 20.3% for DHA (in mg/100 g wet weight), probably due to higher temperature and absorption of other fatty acids from the cooking oil. Combined EPA + DHA levels ranged from 743 to 2223 mg/100 g (wet weight), with smoked fish showing the highest values. Vitamin E exhibited substantial losses during grilling but was largely preserved during smoking, whereas astaxanthin was undetectable in the grilled samples. Vitamin D_3_ demonstrated moderate thermal stability. Overall, each cooking method induced distinct quantitative changes driven by moisture loss and changes in the relative proportions of individual fatty acids within the total lipids. Grilling and smoking were the most favorable for retaining long-chain n-3 PUFA and key micronutrients.

## 1. Introduction

Changing dietary preferences and greater awareness of nutritional value over the past few decades have led to increased global interest in seafood consumption. Seafood is generally recognized as a rich source of high-quality proteins [1,2,3] that support growth and tissue maintenance, polyunsaturated omega-3 fatty acids (n-3 PUFA), which contribute to cardiovascular protection and cognitive health [4,5,6,7] and essential vitamins and minerals that regulate key physiological processes [3,8,9].

Global fisheries and aquaculture output reached record levels in 2022, underscoring the need for strengthened and expanded strategies to ensure that aquatic foods continue to support nutrition, food security, and sustainable livelihoods. As of 2022, the global supply of seafood averaged 20.7 kg per capita which accounted for more than a 120% increase for the last 6 decades. This trend has been driven by numerous factors such as expansion of the aquaculture sector and technology, improved logistics, rising incomes and urbanization, dietary recommendations, etc. [10]. In Bulgaria, seafood is often perceived as more expensive than meat, especially fresh or imported varieties. However, growing recognition of the benefits of n-3 PUFAs and lean proteins is gradually increasing demand. Despite this, seafood intake in Bulgaria, particularly fish products, remains below the EU average and FAO recommendations [11].

Bluefish (*Pomatomus saltatrix* L.) are fast-growing, pelagic predators widely distributed in temperate and subtropical waters, including the Black Sea. They are characterized by a streamlined body, sharp conical teeth, and a voracious feeding behavior, often forming large loose schools that prey on both wild and farmed fish. The species undertakes a spawning migration from the Mediterranean through the Aegean Sea into the Black Sea during spring, followed by a southward return in early autumn [12]. Bluefish has been one of the key commercial and recreational species in the Black Sea and one of the most widely consumed and studied native species along the Bulgarian Black Sea coast [13,14], consistently ranking second in catch volume after European sprat (513.2 metric tons in 2022) [11]. The species is highly valued by local population as a traditional seasonal delicacy due to the rich, distinctive taste. Along the Bulgarian Black Sea coast, bluefish are commonly offered in both fresh and processed forms, with cooking techniques such as grilling, frying, smoking, marinating, and canning playing a central role in enhancing flavor, improving digestibility, and extending shelf life. Cooking influence the retention or loss of key nutrients, making them an important factor in the overall dietary contribution of the species [15,16,17,18,19,20,21].

Thermal processing is a major technological factor that can significantly affect the lipid composition of fish products. Processes such as baking, frying, and smoking alter both the total lipid content and the ratios of the main classes of fatty acids: saturated (SFA), monounsaturated (MUFA), and polyunsaturated fatty acids (PUFA). The extent and nature of these changes depend on the temperature, duration, and heating environment (e.g., air and type of cooking oil). These conditions stimulate chemical reactions, such as the oxidation of unsaturated fatty acids, isomerization, and other transformations, which can lead to both quantitative losses and structural changes in fatty acids, especially the biologically important n-3 PUFAs [16,17,19,20]. According to Tan et al. [17] and Tsoupras et al. [21], thermal processing, particularly frying and smoking, significantly reduces the levels of docosahexaenoic acid (DHA) and eicosapentaenoic acid (EPA) by up to 20–40% compared to raw fish. The authors suggested that this reduction is mainly caused by the oxidation and degradation of polyunsaturated fatty acids, which reduces the nutritional value of marine lipids and can lead to the formation of harmful oxidized by-products. At the same time, the relative content of saturated and monounsaturated fatty acids often increases owing to their higher thermal stability [20]. To preserve the beneficial properties of fish lipids, several researchers have recommended gentle processing methods such as steaming and baking at lower temperatures [17,20]. These techniques can minimize the oxidation and degradation of n-3 PUFAs, thereby preserving their biological activity and benefits to human health.

Although bluefish is an economically important species with great nutritional potential, studies examining the combined effects of grilling, pan-frying, and smoking on its chemical composition, fatty acid profile, fat-soluble vitamins, antioxidant pigments, and cholesterol content are scarce. To fill this gap, the present study provides a comprehensive assessment of how traditional culinary practices affect the nutritional quality of this Black Sea species. The findings offer practical information on nutrient retention and support informed dietary choices based on method-specific changes in fillet composition.

## 2. Materials and Methods

### 2.1. Sample Material Collection

A total of 40 fresh bluefish specimens were purchased from local fishermen as soon as they arrived in Varna fishing seaport in November 2024 and were immediately transported to the laboratory in ice boxes. Fish samples were rinsed with tap water and Milli-Q water system (Millipore, Bedford, MA, USA) and carefully dried with paper towels. Mean weight (79.68 ± 4.73 g) and length (21.98 ± 5.31 cm) were measured using technical scale and digital caliper. Samples were divided into 4 batches (10 fish each): one batch with raw samples and three for the cooking treatments.

### 2.2. Cooking Methods and Sample Preparation

All cooking procedures were performed on whole fish under controlled laboratory conditions, using fixed time and temperature settings specific to each method summarized in Table 1.

Pan frying. Whole fish samples were fried for 5 min in sunflower oil (linoleic acid: 56.8%; oleic acid: 32.1%; palmitic acid: 6.0%; vitamin E: 23 mg/100 g) at 160 °C in a domestic wok pan. Each fish was fried for 3 min on one side and 2 min on the other. After frying, fish were placed on a wire rack lined with paper towels to drain the excess oil.

Grilling. Whole fish samples were grilled for 4–6 min on both sides at 60 °C on a preheated grill. The cooked fish were then placed on a wire rack lined with paper towels for 5 min to rest.

Smoking. Whole fish samples were arranged on smoking trays. The trays were then placed in the smoking oven. The fish were slowly warmed at 40 °C for 2 h and 30 min and then cooked at 90 °C for 8 h. Smoked fish were then let dry at 50 °C for 2 h.

Raw fish were first eviscerated and headed using a PTFE-coated stainless-steel knife, and only the muscle tissue was taken for analysis. For cooked samples, fish were eviscerated prior to cooking and processed whole. After cooking, heads were removed and the muscle tissue was collected. Subsequently, the samples were homogenized (IKA MultiDrive) and used for analysis.

### 2.3. Proximate Composition

The homogenized fish tissue (2.000 ± 0.005 g) was dried at 105 ± 2 °C in an air oven for 18–20 h to a constant weight [22]. The crude protein content was measured using an automated Kjeldahl method, which measures total nitrogen and converts it to protein content using the standard nitrogen-to-protein factor (6.25) [23]. Total lipid content was determined by accelerated solvent extractor (Thermo Scientific Dionex ASE 350, Thermo Fisher Scientific, Waltham, MA, USA) with a mixture of hexane:acetone. The sample (4 g) was homogenized with an equal weight dispersant (8 g) of Dionex™ ASE™ Prep DE (Thermo Fisher Scientific, Waltham, MA, USA) in a mortar and was filled into the 66 mL extraction cell. Optimized ASE parameters: ratio 4:1, *v*/*v* n-hexane/acetone, 80 °C, 1500 psi, 10 min static time, two cycle extraction, and 90% rinse volume). Total extraction time and total solvent volume per sample: ~30 min and ~100 mL, respectively. The extracts were collected in 250 mL vials and were treated with anhydrous sodium sulfate to remove any possible humidity. After filtration, the organic extract was evaporated to dryness on a rotary vacuum evaporator (Hei-Vap Precision Heidolph, Heidolph Instruments GmbH & CO. KG, Schwabach, Germany). Total lipid content of each sample was measured gravimetrically.

### 2.4. Analysis of Nutrients

#### 2.4.1. Fatty Acids Composition

For the analysis of fatty acid composition, lipids were extracted according to the Bligh and Dyer method [24] with some modifications as previously described [18]. Fish tissue homogenates (3 g) were extracted sequentially with CHCl_3_/CH_3_OH (1:2, *v*/*v*), CHCl_3_/CH_3_OH (1:1, *v*/*v*), and CHCl_3_ with constant mixing for 30 min after each extraction step. The combined organic extracts were treated with NaCl solution in H_2_O (0.9%, *w*/*v*) and centrifuged (3500× *g*, 15 min). After phase separation, the bottom chloroform layer was collected using a Pasteur pipette and filtered through anhydrous Na_2_SO_4_. The lipid extracts were evaporated to dryness on a rotary vacuum evaporator (Hei-Vap Precision Heidolph, Heidolph Instruments GmbH & Co. KG, Schwabach, Germany), reconstituted to 1 mL with hexane, and then frozen and kept at −18 °C for further analysis.

Total lipids extracts were trans-methylated into fatty acids methyl esters (FAMEs) with 1 mL 5% H_2_SO_4_–CH_3_OH in a 70 °C water bath [25]. FAMEs were separated on a Trace™ TR-FAME capillary column (60 m × 0.25 mm × 0.25 μm) using Thermo Fisher Scientific FOCUS—PolarisQ Ion Trap gas chromatograph with mass spectrometry detector (Thermo Fisher Scientific, Waltham, MA, USA). A 1 μL aliquot was introduced into the system using a 10:1 split ratio. Helium served as the carrier gas, maintained at a flow rate of 1.2 mL/min. The oven was programmed with the following temperature profile: an initial hold at 100 °C for 1 min, a ramp of 10 °C/min up to 160 °C, then 5 °C/min to 215 °C with a 6 min hold, followed by a further increase of 5 °C/min to 230 °C, held for 5 min. Identification of FAME peaks was achieved by comparing retention times with certified reference standards (Supelco 37 Component FAME Mix and PUFA No. 3 derived from Menhaden oil). Results were expressed as weight percentages of the total fatty acid content.

#### 2.4.2. Fat-Soluble Vitamins, Astaxanthin and Cholesterol

Fat-soluble vitamins (A, D_3_ and E), astaxanthin and cholesterol were analyzed simultaneously by high-performance liquid chromatography (Thermo Scientific Spectra SYSTEM, Thermo Fisher Scientific, Waltham, MA, USA) equipped with a reversed-phase chromatography column (Synergi Hydro-RP 80A 4μ 250 × 4.6 mm, Phenomenex, Torrance, CA, USA). The extraction of analytes and chromatography conditions are described in detail by Dobreva et al. [26]. The concentrations of fat-soluble vitamins were used to calculate the percentage of the recommended daily intake for adults (%RDI) provided by a 100 g serving of fish:

$$\%RDI=\left(\frac{amount\;of\;vitamin\;(A,\;D_{3}\;or\;E)}{RDI}\right)\times 100$$

#### 2.4.3. Nutritional Quality Indices of Lipids

The nutritional quality of raw and cooked bluefish lipids was assessed by three nutritional indices—the Atherogenicity index (AI) [27]; the Thrombogenicity index (TI) [27] and the Hypocholesterolemic/Hypercholesterolemic ratio (h/H) [28]:

Atherogenicity index (AI)

$$AI=\frac{C12:0+(4\times C14:0)+C16:0}{\sum {PUFA}_{n-6}+\sum {PUFA}_{n-3}+\sum MUFA}$$

Thrombogenicity index (TI)

$$TI=\frac{C12:0+C14:0+C16:0}{\left(0.5\times {PUFA}_{n-6}\right)+\left(3\times {PUFA}_{n-3}\right)+\left(0.5\times MUFA\right)+\left(\frac{{PUFA}_{n-3}}{{PUFA}_{n-6}}\right)}$$

Hypocholesterolemic/Hypercholesterolemic ratio (h/H)

$$h/H=\frac{C18:1n-9+C18:2n-6+C18:3n-3+C20:4n-6+C20:5n-3+C22:6n-3}{C14:0+C16:0}$$

### 2.5. Statistical Analysis

Each analysis was performed in triplicate parallel samples, and mean values were used for statistical calculations. The data were expressed as the mean ± standard deviation. One-way ANOVA, followed by a post hoc Tukey’s test, was used to compare the means between the raw and cooked fish. Statistical analyses were performed using GraphPad Prism 8 software with significance at *p* < 0.05 used for the descriptive statistics).

## 3. Results and Discussion

### 3.1. Proximate Composition

The results of the proximate composition (crude protein, crude fat and moisture) of the raw and cooked bluefish are shown in Table 2.

Cooking significantly altered the proximate composition of bluefish across all treatments. Moisture content decreased in all cooked samples, with the greatest reduction observed in pan-fried fish. Crude protein content varied significantly among cooking methods (*p* < 0.05). Pan-fried fish showed the highest value (63.59 ± 0.94 g/100 g dw), whereas smoked fish exhibited the lowest (55.60 ± 1.38 g/100 g dw). Grilled fish (56.01 ± 1.06 g/100 g dw) did not differ significantly from raw samples (58.62 ± 0.73 g/100 g dw). Total lipid content also increased relative to raw bluefish, by 26% in grilled samples, 69% in pan-fried samples, and 80% in smoked samples.

The observed reduction in moisture is consistent with water evaporation during thermal exposure, which concentrates other constituents in the muscle matrix. Similar increases in protein concentration have been reported in pan-fried fish (*Polypterus bichir bichir*) [29] and are commonly attributed to protein denaturation and reduced water-binding capacity. The rise in lipid content reflects combined effects of moisture loss and, in the case of frying, oil uptake. Frying is known to promote oil absorption through capillary action as moisture rapidly evaporates, creating voids in the tissue which can subsequently retain oil. This mechanism, together with the weakening of protein gel structures during heating, has been extensively described in previous studies [29,30,31].

Although lipid accumulation during cooking is well documented, species with high content of endogenous lipid content may experience loss of fish lipids during grilling or smoking due to migration and the release of lipid-derived degradation or oxidation products [32,33]. In the present study, such losses were not observed, which may be related to the moderate initial lipid content of bluefish. Overall, moisture loss appears to be the dominant driver of nutrient concentration, with pan-frying and smoking producing the most pronounced effects.

### 3.2. Fatty Acids Composition

The results for the fatty acid composition (expressed as percentages of the total fatty acids methyl esters and mg per 100 g wet weight) of raw and cooked bluefish are given in Table 3. Thermal processing of bluefish tissues results in significant changes in the content of the main FA groups.

Saturated fatty acid (SFA) levels decreased, most pronounced during frying (approximately −36%), followed by grilling (−16%) and smoking (−12%) compared to the raw sample. These changes may be due to thermal degradation and leakage of solid lipid fractions, as well as possible hydrolysis and evaporation of some lipids during heating. Similar trends were reported by Candela et al. [34], who observed a decrease in total SFA after frying fish, which was associated with melting and oxidation processes. Monounsaturated fatty acids (MUFA) also decrease during grilling (approximately −30%), but their proportion remains stable or slightly increases during frying and smoking. This is probably due to the absorption of vegetable oils rich in oleic acid from the cooking medium, as demonstrated by Neff et al. [35] and Tokarczyk et al. [36]. These fatty acids are more resistant to thermal autoxidation than polyunsaturated fatty acids, which contributes to their lower losses compared to polyunsaturated fatty acids [36,37]. Polyunsaturated fatty acids (PUFA) undergo the most pronounced alterations. Their relative proportions increased across all cooking techniques compared to the raw state, with the greatest increase observed during frying (+87%) and grilling (+75%). However, this apparent increase does not represent an actual increase in PUFA content but rather results from the incorporation of linoleic acid (C18:2n-6c) present in the cooking oils and the simultaneous reduction in other lipid fractions. Consistent findings were reported by Tan et al. [17] and Biandolino et al. [30], who highlighted that frying enriches the PUFA profile mainly through oil absorption during cooking.

Culinary treatments resulted in substantial loss of short- and medium-chain saturated fatty acids (C12:0–C15:0). Lauric acid (C12:0) was reduced by 11% during grilling and by nearly 50% during frying, while tridecanoic acid (C13:0) was completely lost after frying and smoking. Similar losses of short-chain SFA upon high-temperature processing were also reported by Candela et al. [34], who suggested that deep-frying causes significant destruction of volatile saturated lipids due to oxidative and evaporative losses [34]. Medium-chain saturated fatty acids such as palmitic (C16:0) and heptadecanoic (C17:0) acids, which are the dominant SFA in the raw sample, showed the most significant losses. Dominant SFAs such as palmitic (C16:0) and heptadecanoic (C17:0) acids experienced considerable losses, likely due to lipid melting and leakage from muscle during heating. Palmitic acid (C16:0) decreased by 27%, 42%, and 17%, whereas heptadecanoic acid (C17:0) decreased by 37%, 46%, and 25% during grilling, frying, and smoking, respectively, consistent with previous reports [30,36].

Long-chain SFAs (C18:0–C22:0) were comparatively stable, with minimal variation across cooking methods. Stearic acid (C18:0) shows only slight variations (–2% to –22%), while arachidic (C20:0) and behenic (C22:0) acids show minimal fluctuations (<5%), confirming their higher resistance to oxidation and volatilization. Similar stability of long-chain saturated fatty acids under thermal stress has been observed in studies on mackerel and mullet [17,38]. Interestingly, lignoceric acid (C24:0) was only detected in the pan-fried sample (0.24%), which may reflect a minor exogenous contribution from the frying oil or potential chain elongation during heating. In summary, a clear differentiation in thermal changes was observed between individual saturated fatty acids in bluefish. Short-chain SFA were most vulnerable to heat-induced degradation and volatilization. Palmitic (C16:0) and heptadecanoic (C17:0) acids were reliable indicators of lipid loss, while long-chain SFA (C18:0–C22:0) retained their integrity under all cooking conditions. The most pronounced changes occurred during frying, while smoking was the most sparing method for the saturated fatty acids fraction. These findings are consistent with previous studies showing that frying promotes oxidative degradation of saturated and monounsaturated fatty acids through lipid exudation and thermal oxidation [17,30,34,36].

Monounsaturated fatty acids (MUFA) represent the second most abundant fatty acid group in raw bluefish. Their relative proportion decreased following all treatments, with grilling causing the greatest reduction (~30%), whereas frying and smoking resulted in more moderate decreases of about 15% and 12%, respectively. Among the individual MUFAs, palmitoleic acid (C16:1) exhibited the highest sensitivity to heat, with losses of approximately one third during grilling, more than half during frying, and about one sixth during smoking, indicating a pronounced thermal sensitivity. In contrast, oleic acid (C18:1n-9c) followed a different trend: its proportion decreased significantly during grilling and slightly during smoking but increased by nearly 10% in pan-fried samples. This increase is consistent with the partial absorption of sunflower oil, whose fatty acid composition includes relatively high levels of this fatty acid, as well as the relative changes accompanying moisture loss. Minor MUFAs (C20:1 and C22:1) also decreased under all treatments. Overall, grilling resulted in the most pronounced changes in the MUFA profile, frying modified the distribution of individual MUFAs primarily through the combined effects of heating and cooking oil absorption, whereas smoking resulted in the least change compared to the pattern in the raw samples. These results are consistent with previous findings, indicating that MUFAs are moderately stable during thermal processing but undergo compositional changes depending on the heating conditions and duration of exposure [17,30,36].

Polyunsaturated fatty acids (PUFA) constitute an important group of fatty acids in bluefish, supplying nutritionally important n-3 and n-6 fatty acids. Thermal processing caused a significant proportional increase in total PUFA content, with an increase of approximately 75% during grilling, 87% during frying, and 40% during smoking. These changes reflect a redistribution of composition rather than improved preservation of endogenous PUFA. The increases observed after grilling and smoking were mainly due to moisture loss, which concentrated the lipid components, while frying additionally incorporated significant amounts of linoleic acid (C18:2n-6c) from sunflower oil, increasing the apparent proportion of polyunsaturated fatty acids (PUFA). Thus, the final PUFA profile of cooked bluefish was strongly influenced by both processing temperature and cooking environment, with frying leading to the most pronounced changes and smoking the least. Linoleic acid (C18:2n-6c) showed the most pronounced increase within the PUFA group, rising by approximately 70% during grilling and by more than six-fold during frying, reflecting a strong contribution from the sunflower oil used in this treatment. Arachidonic acid (C20:4n-6, ARA) decreased substantially across all treatments, showing an approximate 50% reduction during grilling and more moderate decreases—on the order of 20–30%—during frying and smoking. Eicosapentaenoic acid (C20:5n-3, EPA) remained relatively stable under grilling and smoking, with reductions not exceeding 10–15% but decreased by approximately 40% during frying. Docosahexaenoic acid (C22:6n-3, DHA) displayed higher proportional values after grilling and smoking, increasing by roughly 150% and 100%, respectively, whereas frying resulted in an overall reduction. These changes led to a higher n-6/n-3 ratio in all cooked samples, most pronounced in the pan-fried fillets.

The pronounced rise in linoleic acid (C18:2n-6c), especially during frying, reflects substantial incorporation of sunflower oil into the fillets, consistent with earlier findings that frying oil can markedly elevate the n-6 fraction in fish lipids [17,30]. In contrast, the notable reduction in arachidonic acid (C20:4n-6, ARA) (≈50% during grilling) confirms its susceptibility to high temperatures, aligning with reports of rapid degradation in highly unsaturated n-6 fatty acids [39]. Long-chain n-3 PUFA demonstrated distinct responses to thermal processing. Eicosapentaenoic acid (C20:5n-3, EPA) remained largely unchanged during grilling and smoking (minor reductions <15%) but showed a marked decline during frying (≈40%), which is consistent with evidence that EPA (C20:5n-3) is highly sensitive to oxidation under high-temperature oil exposure [38,40]. Docosahexaenoic acid (C22:6n-3, DHA) increased proportionally after grilling and smoking (approximately 1.5-fold and 2-fold, respectively); however, these apparent increases are best interpreted as concentration effects caused by water loss rather than improved thermal stability. Similar patterns have been observed in other marine species under comparable processing conditions [38,40].

Combined, these shifts resulted in an elevated n-6/n-3 balance after cooking, particularly in pan-fried samples where the strong linoleic acid enrichment coincided with reductions in long-chain n-3 PUFA. This change reflects a decrease in lipid nutritional quality, consistent with previous observations of frying-induced shifts in PUFA profiles [17,30]. Grilling preserved a comparatively balanced profile, while smoking produced intermediate effects. These outcomes highlight that both temperature intensity and cooking medium play a decisive role in shaping PUFA stability and distribution.

Thermal processing significantly influenced the nutritional quality indexes of bluefish lipids, with distinct effects depending on the cooking method (Table 4).

The proportion of n-3 PUFA increased during grilling and smoking, with relative rises of approximately 95% and 50%, respectively, compared with raw samples, reflecting concentration effects due to moisture loss. In contrast, frying led to a decrease of about 35% in n-3 PUFA. The n-6 PUFA fraction showed the opposite trend: it increased strongly during frying (approximately +85%), while grilling and smoking resulted in moderate increases linked to water loss. Consequently, the n-6/n-3 ratio rose in all processed samples and was highest in the pan-fried fillets.

The PUFA/SFA ratio and the AI, TI and h/H indices showed numerical improvements after frying and, to a lesser degree, after grilling and smoking. However, these changes coincided with substantial increases in dietary linoleic acid sourced from the frying oil. EPA + DHA content differed considerably between methods: the highest value was recorded in smoked samples (2223 mg/100 g), followed by grilled (1478 mg/100 g), while pan-fried samples contained the lowest amount (743 mg/100 g).

Compared with raw samples, the increases in n-3 PUFA observed in grilled and smoked fillets are primarily explained by moisture loss, which concentrates the existing lipid fraction. The more moderate rise during smoking reflects its lower processing temperature and longer duration, conditions that typically limit the extent of thermal alteration. These patterns align with the observations of Tan et al. [17], who reported that moderate-temperature treatments tend to preserve the general PUFA distribution more effectively than high-temperature methods.

The strong increase in n-6 PUFA relative to raw fish during frying—approximately +85%—reflects the uptake of linoleic-acid-rich sunflower oil, consistent with findings reported by Biandolino et al. [30]. This shift occurred simultaneously with reductions in EPA and DHA compared with raw samples, particularly during frying, confirming the susceptibility of long-chain n-3 PUFA to high-temperature, oil-rich environments as noted previously [38,40].

As a result, the n-6/n-3 ratio increased substantially relative to raw fish, especially in pan-fried samples, where linoleic acid enrichment coincided with marked losses of EPA and DHA. Grilling produced a more balanced composition relative to raw fish, while smoking showed intermediate results, reflecting its lower temperature and prolonged exposure. The comparison with the raw baseline clearly shows that the highest-temperature method (frying) produced the largest deviation from the native PUFA pattern.

Although PUFA/SFA and related lipid indices (AI, TI, h/H) improved numerically compared with raw fish, these changes largely reflect external enrichment with unsaturated fatty acids rather than genuine preservation of endogenous long-chain n-3 PUFA—an interpretation consistent with previous observations by Tan et al. [17] and Biandolino et al. [30].

Overall, comparison with raw samples demonstrates that temperature, duration, and oil contact collectively determine the magnitude of PUFA modification. Grilling and smoking retain a closer resemblance to the native fatty acid composition of bluefish, whereas frying produces the greatest divergence from the raw profile due to combined effects of high heat and oil absorption.

The observed variations in fatty acid composition and the related nutritional indices indicate that the type and intensity of thermal processing have a measurable impact on the lipid profile of bluefish. As noted by Dobreva et al. [16], moderate cooking methods such as grilling and smoking tend to maintain the relative proportions of the major fatty acid groups (SFA, MUFA and PUFA) and limit the reduction in long-chain n-3 PUFA. The internal balance between saturated and unsaturated fatty acids, including the n-3/n-6 ratio, is an important indicator of the overall nutritional quality and oxidative sensitivity of the lipid fraction. More intensive treatments, particularly frying, produced the largest deviations from the raw lipid profile, characterized by decreases in long-chain n-3 PUFA and increases in MUFA and n-6 PUFA due to oil absorption. As a result, indices such as PUFA/SFA and n-3/n-6 changed in ways that reflect both thermal effects and the contribution of the cooking medium. Apparent improvements in AI, TI and h/H values mainly represent the influence of absorbed unsaturated fatty acids rather than preservation of native lipids, as also discussed by Tan et al. [17]. Overall, the data show that processing temperature, duration and contact with external fat collectively determine the extent of modification within the fatty acid profile, with grilling and smoking causing smaller shifts than frying.

When comparing the present results with other commonly consumed marine species, the EPA + DHA content of raw bluefish is within the range reported for red mullet and salmon, which are well-known sources of long-chain n-3 polyunsaturated fatty acids [30,33]. After heat treatment, smoked bluefish retained 2223 mg/100 g EPA + DHA, values comparable to and even exceeding those observed for salmon and red mullet under similar conditions [30,33,39]. In contrast, pan-fried samples showed the lowest retention of EPA and DHA, consistent with findings for fried salmon, for which losses have also been reported [33,39,40]. The PUFA/SFA and AI/TI values of cooked bluefish remained within the nutritionally favorable ranges previously established for oily fish species such as salmon [33,40]. The comparisons indicate that despite the specific changes observed, bluefish maintains a competitive nutritional profile compared to other marine species traditionally recommended as high-omega-3 foods.

### 3.3. Fat Soluble Vitamins, Astaxanthin and Cholesterol Content

The observed alterations in the content of fat-soluble vitamins, astaxanthin, and cholesterol in bluefish (Pomatomus saltatrix) following thermal processing (Table 5) are attributable to the inherent chemical properties and thermal stability of these compounds under heat and oxidative stress.

Retinol (vitamin A) is highly susceptible to oxidative degradation when exposed to elevated temperatures and oxygen, resulting in approximately 57% loss during grilling, as documented by Dobreva et al. [16] and Hosseini et al. [44]. Alpha-tocopherol (vitamin E), while relatively more stable, undergoes significant thermal degradation during grilling and frying, caused by heat-induced molecular breakdown and oxidation; however, its concentration remains stable or increases slightly during smoking due to the presence of phenolic antioxidants in smoke that inhibit oxidation, as reported by Dobreva et al. [16] and Niki [45]. Although cooking oil is naturally rich in vitamin E, our results showed that the vitamin E content in bluefish muscle decreased from 1.18 mg/g lipid (27.1 mg/100 g dw) in raw samples to 0.56 mg/g lipid (22.9 mg/100 g dw) after frying. This suggests that tocopherols migrate into the frying medium or undergo oxidative degradation, leading to reduced retention in the fish tissue. Such findings are consistent with previous reports indicating that fat-soluble compounds, including carotenoids and tocopherols, are prone to transfer into the oil during frying, thereby lowering their levels in the cooked product [46,47,48]. Vitamin D_3_ exhibits greater thermal stability with moderate reductions under grilling and better preservation under frying and smoking conditions, consistent with findings by Sridonpai [49]. Astaxanthin, a carotenoid antioxidant, is largely degraded under grilling due to high temperature and oxygen exposure but retains about 65–70% of its initial concentration in the lipid fraction during frying and smoking, owing to the protective lipid matrix and milder thermal effects, as described by Ambati et al. [50]. Cholesterol levels increase significantly after grilling due to water loss leading to concentration effects, whereas frying and smoking induce decreases of approximately 15–28% in the lipid fraction, attributed to oxidative degradation and the formation of potentially harmful cholesterol oxidation products, as discussed in Tan et al. [17] and Dantas et al. [51]. From a nutritional perspective, smoked bluefish delivers over 160% of the recommended daily intake (RDI) for vitamin E and 40–50% of the RDI for vitamin D_3_ per 100 g serving, providing significant antioxidant support and immune function enhancement [42]. Consequently, smoking emerges as the preferred thermal processing method for conserving bioactive compounds in bluefish, while grilling leads to the greatest nutrient losses, an important consideration for optimizing culinary practices to maximize nutritional benefits.

## 4. Conclusions

Thermal processing produced clear but method-dependent modifications in the chemical composition of Black Sea bluefish (*Pomatomus saltatrix*). Pan-frying caused the largest deviation from the raw lipid profile due to combined reductions in EPA and DHA and substantial uptake of linoleic acid from the sunflower oil. Grilling induced moderate changes while retaining a comparatively higher proportion of long-chain n-3 PUFA. Smoking resulted in the least alteration, preserving MUFA stability, showing minimal losses of vitamins D_3_ and E, and yielding the highest EPA + DHA content. Across all treatments, bluefish maintained favourable lipid-based nutritional indices and remained a valuable source of long-chain n-3 PUFA and fat-soluble vitamins. Overall, grilling and smoking best preserved the native biochemical characteristics of bluefish, whereas frying produced the most pronounced compositional shifts due to high heat and oil absorption.

The main limitation of this study is the absence of calculated true retention values. Although the presented data provide meaningful insights into nutrient changes across cooking methods, the lack of true retention analysis restricts the ability to fully quantify nutrient preservation relative to the raw material. Nevertheless, despite this limitation, the findings offer practical information on how common household cooking methods influence nutrient retention in bluefish and may support more informed choices when preparing this species, particularly when the aim is to preserve its naturally beneficial lipid profile. Future studies could focus on quantifying oil absorption during cooking and identifying lipid-derived compounds with potential health implications, thereby providing a more complete picture of the biochemical changes in bluefish meat after cooking. Of particular interest is the expansion of investigations to include sensory quality, digestibility, and consumer-oriented outcomes, which will enhance understanding of the nutrient profile of bluefish and strengthen its contribution to nutritional science and public health.

## Figures and Tables

**Table 1:** Parameters of cooking treatment of bluefish.

	Raw	Pan Fried	Grilled	Smoked
Cooking temperature	-	160 °C	60 °C	Pre-heating: 40 °C Cooking: 90 °C Drying: 50 °C
Cooking time	-	2–3 min per side	4–6 min per side	Pre-heating: 2 h 30 min Cooking: 8 h Drying: 2 h
Cooking methods and equipment *	-	Stir frying Wok pan	Blackstone^®^ Griddle	Bradley^®^ Smoker

* parameters were selected in accordance with published data and standard culinary practices to ensure reproducibility and comparability across treatments.

**Table 2:** Proximate composition of raw and cooked bluefish (*Pomatomus saltatrix* L.).

	Raw	Grilled	Pan Fried	Smoked
Crude protein (g/100 g dw)	58.62 ± 0.73 ^b^	56.01 ± 1.06 ^ab^	63.59 ± 0.94 ^c^	55.60 ± 1.38 ^a^
Total lipids (g/100 g dw)	22.96 ± 0.34 ^a^	29.02 ± 0.16 ^b^	38.84 ± 0.98 ^c^	41.40 ± 1.43 ^d^
Moisture	66.73 ± 0.75 ^c^	59.45 ± 0.32 ^b^	54.39 ± 0.45 ^a^	60.53 ± 0.23 ^b^

Values in a row not sharing common superscript are significantly different (*p* < 0.05).

**Table 3:** Fatty acid profiles of raw and cooked bluefish, expressed as percentage of total FA, with absolute values (mg per 100 g wet weight edible portion) shown in brackets.

	Raw	Grilled	Pan Fried	Smoked
C12:0	0.44 ± 0.01 ^b^ (22.8)	0.39 ± 0.05 ^b^ (16.4)	0.22 ± 0.04 ^a^ (14.0)	nd
C13:0	0.38 ± 0.01 ^b^ (19.5)	0.20 ± 0.02 ^a^ (8.34)	nd	nd
C14:0	1.88 ± 0.20 ^b^ (96.4)	3.88 ± 0.05 ^d^ (160.8)	1.50 ± 0.14 ^a^ (97.1)	3.21 ± 0.14 ^c^ (247.9)
C15:0	1.08 ± 0.03 ^b^ (55.5)	0.78 ± 0.03 ^a^ (32.3)	0.82 ± 0.19 ^a,b^ (52.9)	0.85 ± 0.06 ^a,b^ (65.6)
C16:0	26.83 ± 0.39 ^d^ (1373.1)	19.50 ± 0.56 ^b^ (809.0)	15.58 ± 0.57 ^a^ (1011.5)	22.37 ± 0.48 ^c^ (1729.2)
C17:0	1.88 ± 0.05 ^d^ (96.01)	1.19 ± 0.04 ^b^ (49.3)	1.01 ± 0.09 ^a^ (65.5)	1.40 ± 0.05 ^c^ (108.4)
C18:0	6.18 ± 0.18 ^b^ (316.4)	6.20 ± 0.10 ^b^ (257.3)	4.84 ± 0.14 ^a^ (314.1)	6.01 ± 0.15 ^b^ (464.4)
C20:0	0.97 ± 0.01 ^a^ (49.6)	0.94 ± 0.08 ^a^ (38.9)	0.80 ± 0.11 ^a^ (52.1)	0.95 ± 0.08 ^a^ (73.3)
C21:0	0.57 ± 0.01 ^a^ (29.3)	0.52 ± 0.05 ^a^ (21.7)	0.47 ± 0.08 ^a^ (30.2)	0.53 ± 0.05 ^a^ (40.6)
C22:0	0.91 ± 0.01 ^a^ (46.6)	0.87 ± 0.08 ^a^ (36.1)	0.94 ± 0.08 ^a^ (60.9)	0.86 ± 0.09 ^a^ (66.8)
C24:0	nd	nd	0.24 ± 0.02 (15.8)	nd
SFA	41.13 ± 0.12 ^c^ (2105.2)	34.47 ± 0.93 ^b^ (1430.2)	26.41 ± 1.10 ^a^ (1714.3)	36.17 ± 1.04 ^b^ (2796.3)
C14:1	0.49 ± 0.00 ^b^ (25.3)	0.45 ± 0.04 ^a,b^ (18.5)	0.31 ± 0.11 ^a^ (20.2)	0.45 ± 0.03 ^a,b^ (34.7)
C15:1	0.64 ± 0.02 (32.8)	nd	nd	nd
C16:1	8.25 ± 0.07 ^d^ (422.4)	5.36 ± 0.14 ^b^ (222.3)	3.82 ± 0.38 ^a^ (248.0)	6.99 ± 0.15 ^c^ (540.5)
C18:1n-9t	0.79 ± 0.01 ^b^ (40.7)	nd	0.27 ± 0.03 ^a^ (17.7)	0.32 ± 0.03 ^a^ (25.0)
C18:1n-9c	22.66 ± 0.98 ^b,c^ (1159.6)	17.01 ± 0.58 ^a^ (705.9)	24.72 ± 0.79 ^c^ (1604.7)	21.28 ± 0.84 ^b^ (1645.2)
C20:1	1.97 ± 0.08 ^c^ (100.7)	1.20 ± 0.10 ^a^ (49.8)	1.11 ± 0.06 ^a^ (71.8)	1.45 ± 0.04 ^b^ (112.4)
C22:1	0.70 ± 0.03 ^a^ (35.9)	0.64 ± 0.06 ^a^ (26.6)	nd	0.67 ± 0.07 ^a^ (51.7)
C24:1	0.21 ± 0.00 ^a^ (10.7)	0.40 ± 0.02 ^b^ (16.4)	0.17 ± 0.04 ^a^ (10.7)	0.24 ± 0.05 ^a^ (18.8)
MUFA	35.72 ± 0.93 ^c^ (1828.1)	25.05 ± 0.47 ^a^ (1039.4)	30.40 ± 0.89 ^b^ (1973.2)	31.41 ± 0.86 ^b^ (2428.3)
C18:2n-6c	1.12 ± 0.06 ^a^ (57.6)	2.26 ± 0.09 ^a^ (93.8)	28.80 ± 2.86 ^b^ (1869.4)	1.39 ± 0.09 ^a^ (107.4)
C18:3n-3	nd	1.09 ± 0.07 ^a^ (45.2)	0.98 ± 0.17 ^a^ (63.7)	nd
C20:2	0.83 ± 0.02 ^c^ (42.5)	0.33 ± 0.01 ^a^ (13.9)	0.58 ± 0.08 ^b^ (37.9)	0.66 ± 0.04 ^b^ (51.2)
C20:4n-6 ARA	2.46 ± 0.15 ^c^ (125.7)	1.15 ± 0.07 ^a^ (47.8)	1.37 ± 0.14 ^a,b^ (88.9)	1.62 ± 0.11 ^b^ (125.22)
C20:5n-3 EPA	8.40 ± 0.55 ^b^ (430.0)	8.19 ± 0.31 ^b^ (339.9)	4.95 ± 0.45 ^a^ (321.6)	7.92 ± 0.35 ^b^ (612.2)
C22:6n-3 DHA	10.34 ± 0.36 ^b^ (529.1)	27.44 ± 0.72 ^d^ (1138.5)	6.50 ± 0.50 ^a^ (421.6)	20.83 ± 2.01 ^c^ (1610.8)
PUFA	23.15 ± 1.05 ^a^ (1184.8)	40.47 ± 0.66 ^c^ (1679.2)	43.19 ± 1.81 ^c^ (2803.1)	32.42 ± 1.88 ^b^ (2506.9)

Results represent mean values ± standard deviation (n = 3); SFA: saturated fatty acids; MUFA: monounsaturated fatty acids; PUFA: polyunsaturated fatty acids; nd—not detected; values in a row not sharing common superscript are significantly different (*p* < 0.05).

**Table 4:** Nutritional quality of raw and cooked bluefish lipids.

	Raw	Grilled	Pan Fried	Smoked
SFA, mg/100g ww	2105.2 ± 6.18 ^c^	1430.2 ± 38.7 ^a^	1714.3 ± 71.7 ^b^	2796.3 ± 80.8 ^d^
MUFA, mg/100 g ww	1828.1 ± 47.6 ^b^	1039.4 ± 19.6 ^a^	1973.2 ± 57.9 ^c^	2428.3 ± 66.1 ^d^
PUFA, mg/100 g ww	1184.8 ± 53.7 ^a^	1679.2 ± 27.5 ^b^	2803.1 ± 117 ^d^	2506.9 ± 145 ^c^
n-3, mg/100 g ww	959.1 ± 46.6 ^b^	1523.6 ± 32.0 ^c^	806.9 ± 66.3 ^a^	2223.1 ± 162 ^d^
n-6, mg/100 g ww	225.7 ± 9.35 ^b^	155.5 ± 6.79 ^a^	1996.2 ± 171 ^d^	283.9 ± 17.5 ^c^
n-6/n-3	0.24 ± 0.01 ^a^	0.10 ± 0.01 ^a^	2.50 ± 0.42 ^b^	0.13 ± 0.02 ^a^
PUFA/SFA	0.56 ± 0.03 ^a^	1.17 ± 0.05 ^c^	1.64 ± 0.14 ^d^	0.90 ± 0.08 ^b^
AI	0.59 ± 0.01 ^c^	0.54 ± 0.02 ^b^	0.30 ± 0.02 ^a^	0.55 ± 0.02 ^b,c^
TI	0.43 ± 0.01 ^c^	0.22 ± 0.01 ^a^	0.32 ± 0.00 ^b^	0.28 ± 0.02 ^a,b^
h/H	1.57 ± 0.02 ^a^	2.45 ± 0.11 ^b^	3.95 ± 0.26 ^c^	2.08 ± 0.09 ^b^
DHA + EPA, mg/100 g ww	959.1 ± 46.6 ^a^	1478.4 ± 34.8 ^b^	743.2 ± 59.7 ^a^	2223.1 ± 162 ^c^

Results represent mean values ± standard deviation (n = 3); AI: atherogenicity index; TI:, thrombogenicity index [26]; h/H: hypocholesterolemic to hypercholesterolemic ratio [27]; values in a row not sharing common superscript are significantly different (*p* < 0.05).

**Table 5:** Fat soluble vitamins, astaxanthin and cholesterol contents expressed per gram lipid of raw and cooked bluefish (mean ± SD); per 100 g dw shown in brackets and percentage of relative daily intake of vitamins.

	Raw		Grilled		Pan Fried		Smoked	
Vitamin A, μg·g^−1^ lipid	1.5 ± 0.06 ^b^ (34.4)		0.5 ± 0.02 ^c^ (14.5)		1.8 ± 0.05 ^a^ (69.9)		1.6 ± 0.05 ^b^ (66.2)	
Vitamin D_3_, μg·g^−1^ lipid	0.55 ± 0.10 ^a^ (12.6)		0.25 ± 0.03 ^c^ (7.25)		0.42 ± 0.03 ^b^ (16.3)		0.6 ± 0.2 ^a^ (24.8)	
Vitamin E, mg·g^−1^ lipid	1.18 ± 0.07 ^a^ (27.1)		0.36 ± 0.05 ^c^ (10.4)		0.59 ± 0.02 ^b^ (22.9)		1.24 ± 0.09 ^a^ (51.3)	
Astaxanthin, μg·g^−1^ lipid	5.3 ± 0.2 ^a^ (121.7)		nd		3.8 ± 0.3 ^b^ (147.6)		3.5 ± 0.2 ^b^ (144.9)	
Cholesterol, mg·g^−1^ lipid	61.3 ± 0.40 ^b^ (1407.4)		80.0 ± 0.47 ^a^ (2321.6)		44.1 ± 0.29 ^d^ (1712.8)		54.9 ± 0.27 ^c^ (2272.9)	
% RDI	Men	Women	Men	Women	Men	Women	Men	Women
Vit A [41]	1.43	1.64	1.02	1.17	2.98	3.40	3.15	3.60
Vit D_3_ [42]	28.0		27.2		37.5		63.0	
Vit E [43]	60.1	75.1	39.2	49.0	52.1	65.1	130	162

Results represent mean values ± standard deviation (n = 3); RDI: daily reference intake; values in a row not sharing common superscript are significantly different (*p* < 0.05).

## Data Availability

The original contributions presented in this study are included in the article. Further inquiries can be directed to the corresponding author.
